# Improving Adenoma Detection Rates: The Role of the Fecal Immunochemical Test

**DOI:** 10.7759/cureus.14382

**Published:** 2021-04-09

**Authors:** Eugene C Nwankwo, Jefferson Lines, Sahiba Trehan, Michelle Marsh, Amit Trehan, Kuldip Banwait, Srinivas Pathapati, Subhasis Misra, Izi Obokhare

**Affiliations:** 1 Internal Medicine, Saint Louis University, Saint Louis, USA; 2 General Surgery, Texas Tech University Health Sciences Center, Amarillo, USA; 3 Gastroenterology, Amarillo Endoscopy Center, Amarillo, USA; 4 Gastroenterology, Panhandle Gastroenterology, Amarillo, USA; 5 Surgery, Oncology, Texas Tech University Health Sciences Center, Amarillo, USA

**Keywords:** colon cancer prevention, hyperplastic polyp, colonoscopy and polypectomy, health care disparities, adr, quantitative fit test, uninsured patients

## Abstract

Background

There is limited knowledge about adenoma detection rates (ADRs) in patients with a positive fecal immunochemical test (FIT). We hypothesized that colonoscopy performed after FIT would result in higher ADRs.

Methods

We reviewed ADRs for colonoscopies performed after a positive FIT test and compared them to ADR rates for routine colonoscopy performed without an initial FIT test between November 2014 and March 2017 at multiple endoscopy sites.

Results

A total of 979 patients underwent a FIT testing in the Texas panhandle, of whom 12.1% (n=119) tested positive. Also, 32.8% (n=39) were found to have one or more tubular adenomatous polyps on final pathological examination. Among these patients, the majority were female (64.1%; n=25). Of the patients, 15.9% (n=19) had a hyperplastic polyp, 1.7% (n=2) had findings consistent with ulcerative colitis, and 0.8% (n=1) were positive for an adenocarcinoma. In the control group of 2,603 patients in whom routine colonoscopy was performed as the initial tool for screening, 719 were found to have one or more tubular adenomas, with an ADR rate of 27.5%. In this group, the cancer rate was found to be 1%.

Conclusions

There was a significant increase in the ADR when colonoscopy is conducted after a positive FIT test. Recommending colonoscopies after a positive FIT test will not only improve ADRs significantly but also lower the overall healthcare cost for screening colon cancer in this era of escalating healthcare costs.

## Introduction

Colorectal cancer (CRC) is the second leading cause of cancer-related deaths in the United States [1]. The United States Preventive Services Task Force (USPSTF) has given a grade A recommendation to screening for CRC in adults aged 50 to 75 years using fecal occult blood testing, flexible sigmoidoscopy, or colonoscopy [2]. Compared with other ethnic groups, African-Americans are diagnosed with CRC at a younger age and those with CRC have reduced survival rates. The American College of Gastroenterology (ACG) recommends that screening begins at the age of 45 years for this group. Despite this recommendation, CRC screening rates for underserved populations tend to be low. Of adults aged 50-75 years, 62.6% are up to date with their CRC screening, but among uninsured patients, it is only 25.1% [3].

Of the screening methods recommended by the USPSTF, fecal occult blood testing is the least expensive [4]. This makes it a popular choice for large population-based screening programs. However, less than half of patients with normal results return for repeat testing [5,6] and less than 60% with an abnormal result complete a follow-up colonoscopy [7,8]. Reasons given by patients for the lack of compliance in a National Health Service (NHS) CRC screening program using guaiac fecal occult blood testing (gFOBT) included discomfort with handling feces, fear of a positive test result, and believing they were in good health. Educating family and peers about CRC screening was associated with increased compliance [9].

Stool can be tested for occult blood using either gFOBT or fecal immunochemical test (FIT), which uses antibodies against hemoglobin to detect occult blood. In a recent article, Katsoula et al. discussed the diagnostic accuracy of the FIT for CRC screening. In a meta-analysis of 6,204 participants, FIT was found to have an average sensitivity of 93% and specificity of 91%. This yielded a positive likelihood ratio of 10.30 and a negative likelihood ratio of 0.08 [10]. Between the two, FIT is more effective and less costly [11]. Similarly, FIT has a higher positivity and adenoma detection rate (ADR) in uninsured populations when compared with gFOBT [12]. The United States Multi-Society Task Force on Colorectal Cancer (MSTF) recommends using either colonoscopy or FIT as the screening test of choice [13]. FIT has been found to have a higher rate of acceptance than colonoscopies, and mailing FIT kits to patients overdue for screening has been shown to significantly increase screening rates in underserved areas when compared to usual care, colonoscopy invitations, or sending educational materials alone [13-15].

The quality of colonoscopy screening is often measured using ADR, which is the percentage of patients undergoing colonoscopy screening who have one or more adenomas detected. Higher ADR in screening colonoscopy has been associated with lower lifetime risks of CRC and CRC mortality; hence, it is used as a valuable benchmark for grading the quality of screening colonoscopy [16]. We postulated that the ADR for patients who had a positive FIT followed by colonoscopy would be significantly higher than that in patients who had colonoscopies alone.

## Materials and methods

Study design

We conducted a prospective review of retrospective collected data from 10 counties in the Texas panhandle. The study enlisted the help of six experienced endoscopists across four facilities. The endoscopy team included board-certified gastroenterologists and surgeons. This study was designed to evaluate the detection rates of one-time colonoscopy and FIT with subsequent colonoscopy for detecting and diagnosing a variety of adenomas.

Recruitment and data collection

The study started in November 2014 with a robust campaign to improve CRC screening rates among men and women in the panhandle. The campaign includes a total outreach of 1.4 million individuals to date. Means of outreach included 1,436 public education sessions, 8,633 face-to-face contacts, and one million reached via social media sites. It was an endeavor targeting the low income, underserved, and refugee communities within the Texas panhandle. The recruitment period was initiated in May 2015, and data collection from the pilot group was finalized in March 2017.

Patients who showed interest in the study were educated about the importance of screening colonoscopy and the significance of the study. An initial survey was utilized to gather demographic information pertinent to the study (age, gender, ethnicity). Survey responses also elicited information on past medical history and family history including the presence of CRC, adenoma, or inflammatory bowel disease (first-degree relatives with CRC or relatives diagnosed before the age of 60 years) [17,18]. Asymptomatic individuals who were uninsured or underfunded but met the criteria for colorectal screening received initial FIT screening [19]. The exclusion criteria for the study included a personal history of colon cancer or any active gastrointestinal (GI) bleed.

In the study, 979 asymptomatic patients between 39 and 89 years of age underwent FIT testing using the OC-Sensor (Eiken Chemical, Tokyo, Japan), with a cutoff of 10 μg hemoglobin/g feces. This value corresponds to 50 ng hemoglobin/mL buffer followed by a single colonoscopy examination. Of the total respondents, 29 have received prior colonoscopy screenings.

Screening

Our study encompassed collecting the FIT sample and colonoscopy data performed across multiple institutions. Equipment and screening protocols were standardized across all sites.

Prior to the colonoscopy by the endoscopist, bowel preparation and conscious sedation were performed [4]. Polyps were classified by morphology (hyperplastic, pedunculated, or sessile), size, location, and distance from the dentate line. Adenomas were further staged using the standard epithelial histology (glandular dysplasia). The staging was performed based on the classification system of the American Joint Committee on Cancer [20].

FIT consisted of analysis of an adequate stool sample with the use of the automated semi-quantitative OC-Sensor. Patients who indicated a hemoglobin level of at least 75 ng per millimeter were offered a colonoscopy.

Statistical analyses and computation

Initial data were de-identified and aggregated on Microsoft Excel. Data were arranged in a two-way contingency table. We compared ADR rates for colonoscopies after a positive FIT against ADR after colonoscopies without FIT using a similar cohort over a period of 20 months. A p-value of 0.05 was considered statistically significant. Pearson’s chi-squared test of independence and Fisher’s exact test were computed with a confidence interval of 95%.

## Results

Study population and participation

Overall, 979 patients who completed the survey underwent FIT. Of these, 166 (16.9%) patients were found to be FIT-positive. Caucasians made up 56/166 (33.7%) of the cohort. Blacks (African American or Non-Hispanic blacks) comprised 11/166 (6.6%) of the group. Among the 166 positive FIT patients, 119 underwent subsequent colonoscopy. Age of initial participants ranged from 39 to 89 years within all ethnicities. Of the total respondents, 29 had undergone prior colonoscopy screenings. Three patients whose ages were under 45 years and above 75 years were removed from the data analysis.

Diagnostic yield

**Figure 1:**
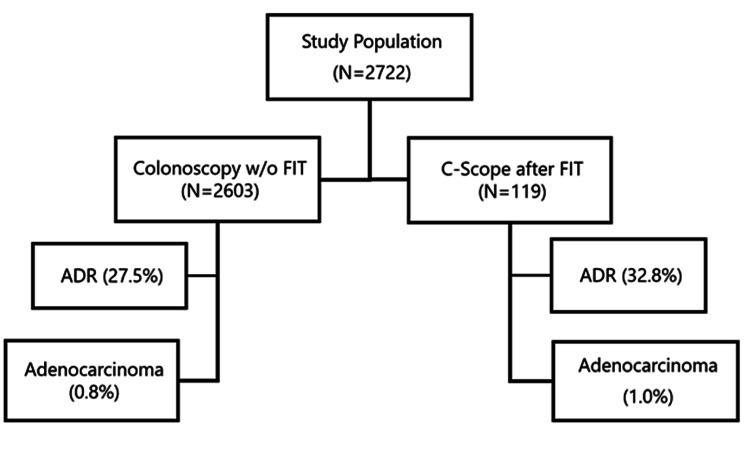
Inclusion criteria for the study included visual and immunochemical evidence of occult lower GI bleed. Patients with a history of inflammatory bowel disease (ulcerative colitis, Crohn’s disease) or prior GI tract carcinoma were excluded from the sample data. There was a higher ADR when FIT testing was employed before a colonoscopy. ADR, adenoma detection rate; GI, gastrointestinal

A total of 39 (32.8%) patients were found to have at least one tubular adenoma (TA) on final pathological examination; 14% were diagnosed with tubulovillous adenomas (TVAs) and another 15.9% were found to have hyperplastic adenomas (HAs). Two (1.7%) patients had findings consistent with ulcerative colitis and another patient (0.8%) was found to have adenocarcinoma (Figure 1). In the control group of 2,603 patients in whom routine colonoscopy was performed as the initial tool for screening, 715 patients were found to have one or more tubular adenomas, with an ADR rate of 27.5%. In this group, the colon cancer rate was found to be 1%.

Detection rates

**Figure 2:**
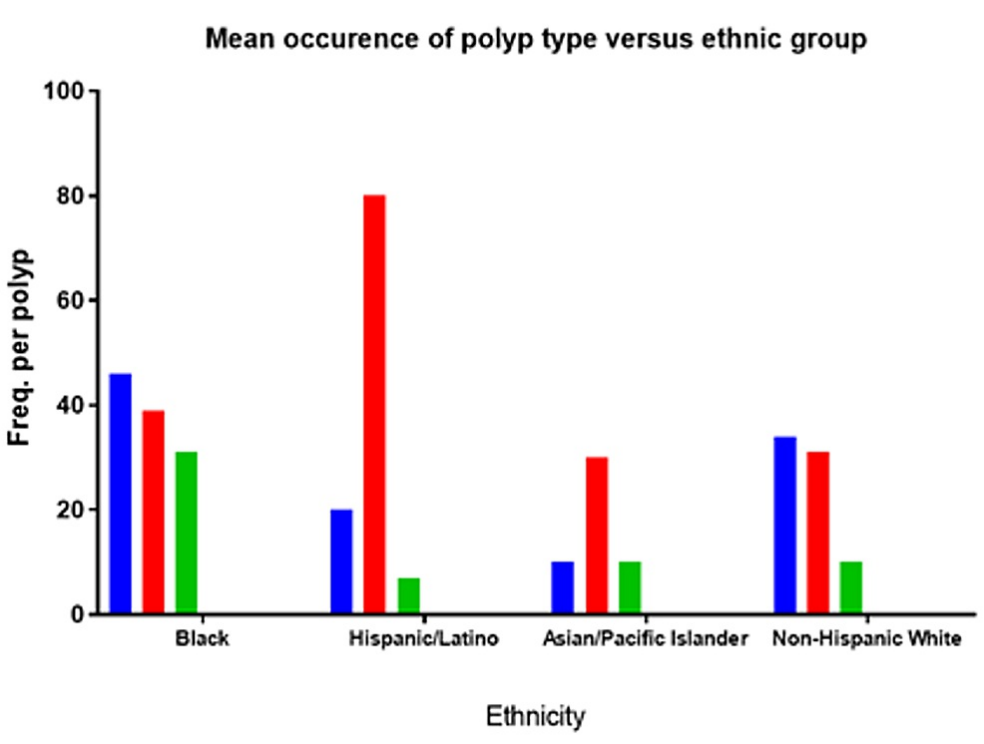
Occurrence of pre-cancerous polyps (tubular adenomas, tubulovillous adenomas) was significantly increased across all ethnic groups compared to benign (hyperplastic polyps). Overall African American/black patients showed significantly higher rates of all polyps regardless of pre-cancerous potential.

Of the total individuals screened with FIT and colonoscopy, there was a mean ADR of 38.7% among Blacks. Within this cohort, the ADR varied slightly between adenoma types. Rates were 46% for HA, 39% for TA, and 31% for TVA. Among Hispanics, the ADR was 20% for HA, 80% for TA, and 7% for TVA. Asian/Pacific Islanders showed an ADR of 10% for HA, 30% for TA, and 10% for TVA (p<0.001) (Figure 2). Overall, the number needed to treat (NNT) for patients who received FIT was 18.9 in comparison to those who only underwent colonoscopy.

Complications and follow-up

Detected polyps were removed using a snare or biopsy forceps. There were no readmissions for post-polypectomy syndrome, bleeding, or perforation. Referrals and treatment were initiated for pathological finding indicative of inflammatory conditions (e.g., ulcerative colitis) or colon cancer. Patients were counseled and scheduled for one-, five-, and 10-year follow-ups based on pathology found during a colonoscopy.

## Discussion

In this prospective review of retrospectively collected data among adults aged 59 to 89 years in the Texas panhandle, we observed that the ADR for patients who had a positive FIT prior to colonoscopy was significantly higher (32.8%) than the ADR for patients who had colonoscopies alone (27.5%) (Table 1) (p<0.0001).

**Table 1:** Overall ADR of sampled cohorts. Colonoscopy only group showed ADR lower than FIT + colonoscopy (27.5% vs. 32.8%). ADR, adenoma detection rate; FIT, fecal immunochemical test

ADR based on type of screening (%)	
Colonoscopy	27.5
FIT + colonoscopy	32.8

Our study has a number of strengths. The outreach program analyzed in this study used multilingual pamphlets and translation services to mitigate language barriers, which might have limited the inclusion of non-English speaking patients. The program’s use of no-cost screening also helped mitigate the negative effects that socioeconomic factors might have on individual participation in the program.

This study also has a number of limitations. First, the generalizability of this study is limited because the cohort is confined to the Texas panhandle. ADR can vary based on several factors, including physician experience, socioeconomic status, and ethnicity [21-23]. Differences in these factors might affect the applicability of our data for other areas of the country. Second, the study has a small sample size. Although the initial population size reached 1.4 million, only 979 patients completed and returned the FIT. The number with a positive FIT during the study period was 166, and the total number undergoing colonoscopy was 119. The sample size for minority ethnicities was particularly low despite the fact that the same ethnicities were overrepresented within our cohort when compared to the general population of the Texas panhandle. Third, this study was also limited by the non-randomized retrospective nature of the trial. We were unable to blind the physicians performing the colonoscopies to the results of their patients’ previous FIT, and this could have introduced an unmeasurable inherent observer bias. The final challenge to this study is the inherent diagnostic accuracy of the FIT test. Various factors would have affected the predictive value of FIT. These factors include, but are not limited to, aspirin, NSAIDs (non-steroidal anti-inflammatory drugs), or anticoagulant use. Patients with active or history of upper and lower GI bleeding were excluded from this study [24].

The American Society for Gastrointestinal Endoscopy (ASGE) and the ACG have recommended using ADR as the number one quality indicator for colonoscopies [21-25]. As of 2015, the ASGE and ACG recommend enforcing an average minimum ADR of 25% (30% for males and 20% for females) as a standard for physicians performing colonoscopies. In this study, the ADR was 27.5% in patients for whom routine colonoscopy was used as the initial screening tool (Table 1). Many factors can affect the ADR in a population undergoing CRC screening, such as the experience of the endoscopist, quality of the prep, and extent or reach of the colonoscopy. The effect of a positive gFOBT or FIT prior to colonoscopy has on ADR has not been well studied.

The variance in ADR between different physicians and the negative effect a low ADR has on CRC screening are the major reasons the ASGE and ACG emphasize the use of ADR as a quality indicator for colonoscopies. Reaching the recommended performance target for ADR was strongly associated with important clinical outcomes. Further prospective double-blind studies elucidating the impact of FIT prior to colonoscopy are vital to reducing the cost of healthcare. Major obstacles of screening large, underserved populations for CRC include the exorbitant cost, the limited availability of qualified endoscopists, and lack of patient education. Screening patients with FIT prior to colonoscopy would positively affect the cost, compliance, and patient comfort of population-based CRC screening programs.

Another major potential benefit of using a positive FIT as a prerequisite for colonoscopy in population-based CRC programs is cost. FIT kits typically cost less than $30, whereas a colonoscopy typically costs more than $2,000 due to the endoscopy and facility fees required. Since population-based CRC screening programs often target the uninsured and underfunded populations, the high costs associated with colonoscopies can limit the extent and reach of those programs. By using a positive FIT as a prerequisite for colonoscopy, CRC screening can reduce the cost of colonoscopies while simultaneously increasing ADR. We would recommend screening high-risk, underfunded patients with an annual FIT followed by colonoscopy when positive.

## Conclusions

ADR is a quality measure for endoscopy facilities and professionals. ADR is defined as “the rate at which a physician finds one or more precancerous polyps during a normal screening colonoscopy procedure for patients over 50 years old.” Professional societies, including the ACG, have determined that the benchmark rate should be at least 25% in men and 15% in women for physicians performing colonoscopies. In this study, we showed a notable difference in detecting precancerous polyps using colonoscopy alone versus FIT before colonoscopy.

Understanding the impact of FIT on ADRs offers advantages that span various aspects of patient care, including reduction of colon cancer mortality and screening costs, therefore increasing access to colon cancer screening for the most vulnerable populations.
